# Atrial fibrillation after cavotricuspid isthmus ablation for isolated typical atrial flutter: contemporary insights from a large global federated research network

**DOI:** 10.1093/europace/euag085

**Published:** 2026-04-16

**Authors:** Laurent Fauchier, Yassine Lemrini, Thibault Lenormand, Arnaud Bisson

**Affiliations:** Service de Cardiologie, Centre Hospitalier Universitaire Trousseau et Faculté de Médecine, Université François Rabelais, Tours 37044, France; Service de Cardiologie, Centre Hospitalier Universitaire Trousseau et Faculté de Médecine, Université François Rabelais, Tours 37044, France; Service de Cardiologie, Centre Hospitalier Universitaire Trousseau et Faculté de Médecine, Université François Rabelais, Tours 37044, France; Service de Cardiologie, Centre Hospitalier Universitaire Trousseau et Faculté de Médecine, Université François Rabelais, Tours 37044, France

**Keywords:** Atrial flutter, Atrial fibrillation, Cavotricuspid isthmus ablation

## Introduction

Cavotricuspid isthmus (CTI) ablation effectively eliminates typical atrial flutter (AFL), yet atrial fibrillation (AF) frequently emerges during follow-up.^1–5^ Reported post-ablation AF rates range from 30% to 70%, depending on patient selection, monitoring intensity, and follow-up duration.^3^ Although combined pulmonary vein isolation (PVI) may reduce the risk of subsequent AF,^6^ contemporary real-world data in strictly AF-naïve patients, describing not only AF incidence but also AF phenotype and subsequent rhythm-control strategies, remain limited. Using a large multicentre electronic health record (EHR) network, we aimed to characterize post-CTI atrial arrhythmias in modern clinical practice.

## Methods

The TriNetX Collaborative Network provides access to de-identified EHRs from 168 healthcare organizations across 18 countries. We identified adults with typical AFL (ICD-10 I48.3) who underwent CTI ablation between 2016 and 2024, excluding those with prior AF, atypical AFL or PVI. Outcomes included paroxysmal, persistent, or permanent AF, recurrent atypical AFL, redo CTI ablation, atrioventricular node ablation, and subsequent AF ablation. AF phenotypes were identified using ICD-10 diagnostic codes (paroxysmal AF I48.0, persistent AF I48.1, and permanent/chronic AF I48.2). Mortality corresponded to all-cause death recorded in the TriNetX network based on EHR ‘deceased’ status or diagnostic codes indicating death. Follow-up extended up to 8 years after the index procedure. The number of events and annualized incidence rates were calculated within the TriNetX analytics platform.

## Results

**Figure 1:**
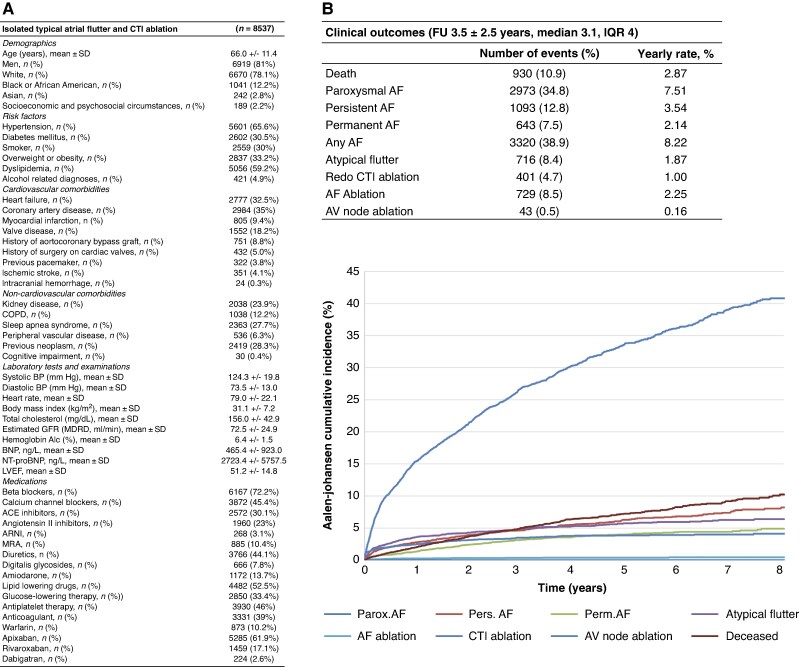
**Baseline characteristics and atrial arrhythmia outcomes after CTI ablation for isolated typical atrial flutter.** (*A*) Baseline characteristics and clinical outcomes of patients with isolated typical atrial flutter treated with cavotricuspid isthmus ablation in the TriNetX network. (*B*) Curves showing the cumulative incidence of clinical outcomes during follow-up after CTI ablation estimated using the Aalen–Johansen method. Values are *n* (%) or mean ± SD. ACE = angiotensin converting enzyme; AF = atrial fibrillation; ARNI = angiotensin receptor-neprilysin inhibitor; BNP = Brain Natriuretic Peptide; BP = blood pressure; COPD = Chronic obstructive pulmonary disease; CTI = cavotricuspid isthmus; eGFR = estimated glomerular filtration rate; ICD = implantable cardioverter defibrillator; LVEF = left ventricular ejection fraction; MRA = mineralocorticoid receptor antagonist; NT-proBNP = N-Terminal Prohormone Brain Natriuretic Peptide; SD = standard deviation.

Among 8537 adults with isolated typical AFL treated by CTI ablation in 54 contributing centres (age 66.0 ± 11.4 years, 81% men), hypertension (66%), dyslipidaemia (59%), diabetes (31%), coronary artery disease (35%), and heart failure (33%) were the most frequent comorbidities, while 9% and 5% had a history of coronary surgery or valve surgery, respectively. Of note, 4.1% had a history of ischaemic stroke at the time of CTI ablation (*Figure 1A*). During a follow-up of 3.5 ± 2.5 years (median 3.1), AF occurred in 38.9% of patients (8.2% per year), including 34.8% with paroxysmal AF (7.5% per year), 12.8% with persistent AF (3.5% per year), and 7.5% with permanent AF (2.1% per year). Atypical flutter occurred in 8.4% of patients, while redo CTI ablation, AF ablation, and AV node ablation were performed in 4.7%, 8.5%, and 0.5%, respectively. Overall mortality was 2.9% per year (*Figure 1B*).

## Discussion

CTI ablation remains the treatment of choice for typical AFL, with high acute success and low recurrence, but AF frequently emerges during follow-up, reflecting the continuum of atrial remodelling.^7–9^ In this large cohort, we provide contemporary estimates of arrhythmia outcomes after CTI ablation. During follow-up, one-third of patients developed AF, most often in its paroxysmal form. Beyond confirming that AF is common after CTI ablation, this study provides contemporary real-world benchmarks for AF phenotype distribution and downstream rhythm-control strategies in a large strictly AF-naïve population, an aspect inconsistently addressed in prior studies.

Earlier series described post-ablation AF in 30–50% of patients, whereas continuous monitoring revealed higher rates approaching 70%.^1–3^ Differences likely relate to monitoring intensity, duration of follow-up, and population characteristics. Importantly, our analysis excluded patients with any previous documentation of AF, unlike many earlier studies. It captured only clinically documented AF rather than subclinical episodes (of uncertain clinical significance) detected by continuous monitoring. These distinctions likely contributed to the relatively lower AF incidence observed in our cohort.

The distribution of AF patterns in our cohort (a predominance of paroxysmal AF but substantial proportions of persistent and permanent AF) reinforces the concept of progressive atrial disease. Given the high prevalence of cardiovascular comorbidities, one might have expected a greater burden of persistent AF, in which substrate tends to predominate over triggers. The predominance of paroxysmal AF therefore suggests that even in a comorbid population, AF after CTI ablation may still be largely trigger-driven initially, before structural remodelling leads to more sustained forms over time. Although this analysis was not designed to identify predictors of AF, the large real-world cohort provides clinically relevant estimates of AF burden and phenotype distribution that may inform surveillance strategies and future risk-stratification studies.

These data emphasize the importance of rhythm surveillance and anticoagulation reassessment after CTI ablation, even when AFL appears ‘isolated’. AF ablation was ultimately required in fewer than 8% of patients, and redo CTI procedures were rare, underscoring both the durability of CTI ablation and the predominance of conservative management strategies. This discrepancy between AF occurrence and ablation frequency suggests that many episodes are asymptomatic, paroxysmal, or clinically tolerated, particularly in an older, comorbid population in whom rate control and anticoagulation remain key therapeutic strategies.

Future work might refine risk stratification after CTI ablation by integrating clinical, imaging, or electrophysiological data to identify patients most likely to develop clinically significant AF. The addition of PVI might be best reserved for those with identifiable substrate abnormalities or recurrent symptomatic AF rather than performed systematically.

This study has limitations inherent to retrospective analyses based on EHRs. Diagnoses relied on administrative coding, without external validation. Detailed information on rhythm monitoring (e.g. ECG recordings, Holter monitoring, implanted devices, or systematic outpatient surveillance) was not available in the EHR network. Consequently, arrhythmia detection likely varied according to follow-up intensity and clinical practice, and it is unknown whether AF during follow-up was detected because of symptoms or during routine surveillance. Detailed clinical data such as atrial dimensions or procedural data were unavailable, as well as left or right origin for atypical flutter during follow-up, partly limiting the granularity of the cohort description. Anticoagulation was assessed at baseline only. The modest rate likely reflects heterogeneous anticoagulation practices in isolated AFL despite a moderate estimated CHA_2_DS_2_-VASc score (∼2.7).^10^

## Conclusion

In this large contemporary real-world cohort of patients undergoing CTI ablation for isolated typical AFL, AF developed in about one-third of patients over a mean follow-up of 3.4 years, most often paroxysmal. Subsequent ablation procedures were infrequent, supporting the continued relevance of CTI ablation as a stand-alone strategy for many patients, although the absence of systematic rhythm monitoring may have led to underdetection of asymptomatic AF. These findings provide contemporary real-world benchmarks for post-CTI arrhythmia outcomes and downstream rhythm-control strategies and inform current debates regarding systematic vs. selective use of concomitant PVI in patients with typical atrial flutter.

## Data Availability

Aggregated data supporting the findings are available within the TriNetX network upon reasonable request.
